# Families of proper holomorphic maps

**DOI:** 10.1007/s12220-026-02342-y

**Published:** 2026-02-02

**Authors:** Barbara Drinovec Drnovšek, Jure Kališnik

**Affiliations:** Faculty of Mathematics and Physics, University of Ljubljana, and Institute of Mathematics, Physics, and Mechanics, Jadranska 19, 1000 Ljubljana, Slovenia

**Keywords:** Riemann surface, Proper holomorphic map, Primary 32H35, Secondary 32H02, 53A10

## Abstract

Given a smooth, open, oriented surface *X* endowed with a family of complex structures $\{J_b\}_{b\in B}$ depending continuously on the parameter *b* in a metrisable space *B*, we construct a continuous family of proper holomorphic maps $F_{b}:(X,J_b)\rightarrow \mathbb {C}^{2}$, $b\in B$.

## Introduction

Every smooth, open, oriented surface *X* endowed with an almost complex structure *J* is a Riemann surface. Therefore, by choosing a continuously varying family of almost complex structures $(J_b)_{b\in B}$ for some parameter space *B*, we determine a family of open Riemann surfaces $(X,J_b)_{b\in B}$. In 2025, Forstnerič [7] initiated the study of continuous maps *F* from $B\times X$ to Euclidean space, or more generally, to an Oka manifold, such that for each ${b\in B}$ the map $F(b,\cdot )$ is holomorphic on the Riemann surface $(X,J_b)$. In this framework, he obtained the Runge and Mergelyan approximation theorems, as well as the Weierstrass interpolation theorem.

Our main result answers in part the question raised by Forstnerič [7, Problem 8.7 (a)] concerning the existence of proper holomorphic maps in this setting:

### Theorem 1.1

Let *X* be a smooth, connected, open, oriented surface, *B* a metrisable space, and $\{J_b\}_{b\in B}$ a continuous family of complex structures on *X* of class $C^{(k,\alpha )}$ with $k\in {\mathbb {Z}}_+,0<\alpha <1$. Then there exists a continuous map $F:B\times X\rightarrow \mathbb {C}^{2}$ such that for every $b\in B$ the map $F(b,\cdot ):(X,J_b)\rightarrow \mathbb {C}^{2}$ is proper holomorphic.

Precise definitions will be given in the next section. It is classical that for every open Riemann surface there is a proper holomorphic immersion into $\mathbb {C}^2$ and a proper holomorphic embedding into $\mathbb {C}^3$, see [6, Theorem 2.4.1] and the references therein.

By increasing the dimension of the target Euclidean space by one, we obtain a family of proper holomorphic immersions:

### Corollary 1.2

Let *X* be a smooth, connected, open, oriented surface, *B* a finite CW complex or a smooth manifold, and $\{J_b\}_{b\in B}$ a continuous family of complex structures on *X* of class $C^{(k,\alpha )}$ with $k\ge 1,0<\alpha <1$. Then there exists a continuous map $G:B\times X\rightarrow \mathbb {C}^{3}$ such that for every $b\in B$ the map $G(b,\cdot ):(X,J_b)\rightarrow \mathbb {C}^{3}$ is a proper holomorphic immersion.

### Proof

By [7, Corollary 8.3] there exists a continuous function $h:B\times X\rightarrow \mathbb {C}$ such that $h(b,\cdot ):(X,J_b)\rightarrow \mathbb {C}$ is a holomorphic immersion for every $b\in B$. Let $F:B\times X\rightarrow \mathbb {C}^{2}$ be a continuous map such that for every $b\in B$ the map $F(b,\cdot ):(X,J_b)\rightarrow \mathbb {C}^{2}$ is proper holomorphic, provided by Theorem 1.1. Then the map $(F,h):B\times X\rightarrow \mathbb {C}^{3}$ is continuous and for every $b\in B$ the map $(F,h)(b,\cdot ):(X,J_b)\rightarrow \mathbb {C}^{3}$ is a proper holomorphic immersion. $\square$

We extend to families the result of Forstnerič and Globevnik [8, Theorem 1.4], Alarcón and López [3, Corollary 1.1], and Andrist and Wold [4, Theorem 5.6] on proper harmonic maps from open Riemann surfaces to $\mathbb {R}^{2}$:

### Theorem 1.3

Let *X* be a smooth, connected, open, oriented surface, *B* a metrisable space, and $\{J_b\}_{b\in B}$ a continuous family of complex structures on *X* of class $C^{(k,\alpha )}$ with $k\in {\mathbb {Z}}_+,0<\alpha <1$. There exists a continuous map $H:B\times X\rightarrow \mathbb {R}^{2}$ such that for every $b\in B$ the map $H(b,\cdot ):(X,J_b)\rightarrow \mathbb {R}^{2}$ is proper harmonic.

The proof relies on the proof of Theorem 1.1 and we postpone it to Section 3.

By Remmert’s proper mapping theorem, the image of an analytic subvariety under a proper holomorphic map is an analytic subvariety. Therefore, the following corollary provides, in particular, a path of complex analytic subvarieties in $\mathbb {C}^2$ from the one parametrised by the complex line to the one parametrised by the unit disc.

### Corollary 1.4

Let *X* be a smooth, connected, open, oriented surface. Let $J_0, J_1$ be complex structures on *X* of class $C^{(k,\alpha )}$ with $k\in {\mathbb {Z}}_+,0<\alpha <1$. There exist a continuous family $\{J_b\}_{b\in [0,1]}$ of complex structures on *X* of class $C^{(k,\alpha )}$ and a continuous map $F:[0,1]\times X\rightarrow \mathbb {C}^{2}$ such that for every $b\in [0,1]$ the map $F(b,\cdot ):(X,J_b)\rightarrow \mathbb {C}^{2}$ is proper $J_b$-holomorphic.

### Proof

Each complex structure determines a compatible Riemannian metric on *X* of the same smoothness class. Convex combinations of these metrics yield a path connecting the two, which in turn induces a corresponding path of almost complex structures on *X* of the same smoothness class; see, for example [2, Lemma 1.9.1]. Then the conclusion follows from Theorem 1.1. $\square$

The main idea in the proof is constructing a convergent sequence of maps on an exhausting sequence of Runge compact sets of *X* in a way similar to constructions in [1, 3, 5]. In [3], Alarcón and López constructed a proper conformal minimal immersion from any open Riemann surface *M* into $\mathbb {R}^3$ with its image in a wedge, and in [1], Alarcón and Forstnerič obtained a proper holomorphic immersion from any open Riemann surface *M* into $\mathbb {C}^2$ directed by an Oka cone. The main tool in our construction is the Mergelyan approximation theorem for proper families of compact Runge sets recently proven by Forstnerič [7]. When the parameter space *B* is not compact, one has to deal with nonconstant proper families of compact Runge subsets of *X*, which are present already in the noncritical case, i.e., when the topology of *X* is trivial.

## Preliminaries

We use the notations $\mathbb {N}=\{1,2,3,\ldots \}$ and $\mathbb {Z}_{+}=\{0,1,2,3,\ldots \}$ for the set of natural numbers, respectively the set of nonnegative integers. If *K* is a compact topological space and $f:K\rightarrow \mathbb {C}$ is a continuous function, we denote by $\Vert f\Vert _{K}$ the supremum norm of *f* on *K*.

Throughout the paper, we denote by *X* a smooth, connected, open, oriented, Hausdorff, second countable surface. We are interested in families of complex structures on *X*, parametrised by some topological space *B* as defined in [7]. A complex structure on *X* is given by a section $J\in \Gamma (\text {End}(TX))$ of the bundle of endomorphisms $\text {End}(TX)$ of the tangent bundle *TX* of *X* that satisfies the condition $J^{2}=-\textrm{Id}$. We always assume that *J* induces on *X* the given orientation of *X*. Since the tangent bundle *TX* is trivial, the bundle $\text {End}(TX)$ is isomorphic to the trivial bundle $X\times \text {End}(\mathbb {R}^{2})$. If we choose a trivialisation of $\text {End}(TX)$, we can identify sections of $\text {End}(TX)$ with functions from *X* to $\text {End}(\mathbb {R}^{2})$. If we furthermore choose a Riemannian metric on *X*, we can define Banach spaces $\Gamma ^{(k,\alpha )}(\text {End}(TX)|_{\Omega })$ of sections of $\text {End}(TX)$ of Hölder class $C^{(k,\alpha )}(\Omega )$ for any $k\in \mathbb {Z}_{+}$, $0<\alpha <1$ and any relatively compact domain $\Omega \subset X$. A complex structure $J\in \Gamma (\text {End}(TX))$ is locally of class $C^{(k,\alpha )}$ if $J|_{\Omega }\in \Gamma ^{(k,\alpha )}(\text {End}(TX)|_{\Omega })$ for every relatively compact domain $\Omega \subset X$.

### Definition 2.1

Let *B* be a topological space, $k\in \mathbb {Z}_{+}$ and $0<\alpha <1$. A **continuous family of complex structures on ***X* of class $C^{(k,\alpha )}$, parametrised by *B*, is a family of complex structures $J=\{J_{b}\}_{b\in B}$ on *X*, which are locally of class $C^{(k,\alpha )}$, such that for every relatively compact domain $\Omega \subset X$ the map $b\mapsto J_{b}|_{\Omega }\in \Gamma ^{(k,\alpha )}(\text {End}(TX)|_{\Omega })$ is continuous.

A continuous family $J=(J_{b})_{b\in B}$ of complex structures on *X* furnishes us with a Riemann surface $(X,J_{b})$ for every $b\in B$. A function $f:X\rightarrow \mathbb {C}$ is $J_{b}$**-holomorphic** if it is holomorphic with respect to the complex structure $J_{b}$ on *X* and the standard complex structure on $\mathbb {C}$.

Let *B* be a topological space and let *A* be a subset of $B\times X$. For every $b\in B$ we denote

$$ A_{b}=\{x\in X\,:\,(b,x)\in A\}. $$

If $f:A\rightarrow \mathbb {C}$ is a function and $b\in B$, we denote by $f_{b}:A_{b}\rightarrow \mathbb {C}$ the function, given by

$$ f_{b}(x)=f(b,x) $$

for $x\in A_{b}$. We are interested in continuous families of holomorphic functions.

### Definition 2.2

Let *B* be a topological space, $k\in \mathbb {Z}_{+}$, $0<\alpha <1$ and let $J=(J_{b})_{b\in B}$ be a continuous family of complex structures on *X* of class $C^{(k,\alpha )}$, parametrised by *B*.

(1) Let $U\subset B\times X$ be an open subset. A continuous function $f:U\rightarrow \mathbb {C}$ is *J***-holomorphic**, if the function $f_{b}:U_{b}\rightarrow \mathbb {C}$ is $J_{b}$-holomorphic for every $b\in B$. The vector space of all *J*-holomorphic functions on *U* is denoted by $\mathcal {O}_{J}(U)$. We similarly define a *J*-holomorphic map $f:U\rightarrow M$, where *M* is a complex manifold.

(2) Now let $Z\subset B\times X$ be a closed subset. A continuous function $f:Z\rightarrow \mathbb {C}$ is *J***-holomorphic** if there exist an open set $U\subset B\times X$, containing *Z*, and $\tilde{f}\in \mathcal {O}_{J}(U)$ such that $\tilde{f}|_{Z}=f$. The vector space of all *J*-holomorphic functions on *Z* is denoted by $\mathcal {O}_{J}(Z)$. The vector space of all continuous functions $f:Z\rightarrow \mathbb {C}$, for which the function $f_{b}:\text {Int}\,(Z_{b})\rightarrow \mathbb {C}$ is $J_{b}$-holomorphic for every $b\in B$, is denoted by $\mathcal {A}_{J}(Z)$.

For our construction, we need to consider continuous functions on proper families of compact subsets of *X*, which we recall below.

### Definition 2.3

Let *B* be a topological space and let $\pi :B\times X\rightarrow B$ be the projection onto the first factor. A **family of compact subsets of ***X*, parametrised by *B*, is given by a closed subset $K\subset B\times X$, for which $K_{b}$ is a compact subset of *X* for every $b\in B$ (note that $K_{b}$ may be empty). A family of compact subsets *K* is **proper** if the map $\pi |_{K}:K\rightarrow B$ is proper, it is **wide** if $K_{b}$ is non-empty for every $b\in B$, and it is called **Runge** if $K_{b}$ is Runge for every $b\in B$.

Recall that a continuous map between topological spaces is proper if the preimage of every compact subset is compact, and that a compact subset $K\subset X$ is Runge if the complement $X\setminus K$ has no relatively compact connected components. A Runge compact set *K* is holomorphically convex in every complex structure on *X*.

As noted in [7], we have the following characterization of proper families of compact subsets:

### Proposition 2.4

Let *B* be a Hausdorff topological space and let $K\subset B\times X$ be a closed subset. Then *K* is a proper family of compact subsets of *X* if and only if the following two conditions hold:

(1) For every $b\in B$ the fiber $K_{b}$ is compact,

(2) For every $b_{0}\in B$ and every open subset $U\subset X$ containing $K_{b_{0}}$ there is a neighbourhood $B_{0}$ of $b_{0}$ in *B* such that $K_{b}\subset U$ for every $b\in B_{0}$.

Let us now take a look at some examples.

### Example 2.5

(1) For every compact subset $K_{0}\subset X$ we have the constant family $K=B\times K_{0}$ of compact subsets of *X* for which $K_{b}=K_{0}$ for every $b\in B$. More generally, let $K\subset B\times X$ be a closed subset for which $\pi |_{K}:K\rightarrow B$ is a fiber bundle with a compact fiber. Then *K* is a proper family of compact subsets of *X*.

(2) A proper family of compact subsets of *X* need not have all fibers homeomorphic. As an example, consider the case when $B=\mathbb {R}$, $X=\mathbb {C}=\mathbb {R}^{2}$ and denote by $\overline{\mathbb {D}}\subset \mathbb {C}$ the closed unit disk. The set

$$ K=\left( (-\infty ,0]\times \overline{\mathbb {D}}\right) \cup \left( [0,\infty )\times \{0\}\right) $$

is then a proper family of compact subsets of *X*. On the other hand, let us define the set

$$ \tilde{K}=\left( (-\infty ,0]\times \{0\}\right) \cup \left\{ (x,\tfrac{1}{x})\,|\,x\in (0,\infty )\right\} . $$

The set $\tilde{K}$ defines a family of compact subsets of *X* which is not a proper family.

To show that a given set is a proper family of compact subsets of *X* we easily obtain the following useful criteria.

### Proposition 2.6

Let *B* be a topological space.

1. Let $K\subset B\times X$ be a proper family of compact subsets of *X* and let $K'$ be a closed subset of *K*. Then $K'$ is a proper family of compact subsets of *X* as well.
2. Let $K_{1},K_{2}\subset B\times X$ be proper families of compact subsets of *X*. Then $K_{1}\cup K_{2}$ is a proper family of compact subsets as well.

Let $K\subset B\times X$ be a wide, proper family of compact subsets of *X* and let $\eta :K\rightarrow (0,\infty )$ be a positive continuous function. Since $K_{b}$ is non-empty for every $b\in B$, there exists the minimum

$$ \min (\eta )(b)=\min \{\eta (b,x)\,:\,x\in K_{b}\}>0. $$

We thus obtain a function $\min (\eta ):B\rightarrow (0,\infty )$, which is continuous if *K* is a constant or a locally trivial family. In general, however, the function $\min (\eta )$ is only lower semicontinuous, but we can always find a continuous minorant $m(\eta ):B\rightarrow (0,\infty )$ of $\min (\eta )$:

### Proposition 2.7

Let *B* be a metrisable space, $K\subset B\times X$ a wide, proper family of compact subsets of *X* and let $\eta :K\rightarrow (0,\infty )$ be a continuous function.

1. The function $\min (\eta ):B\rightarrow (0,\infty )$ is lower semicontinuous.
2. There exists a continuous function $m(\eta ):B\rightarrow (0,\infty )$, such that $m(\eta )(b)<\min (\eta )(b)$ holds for every $b\in B$. If *B* is a smooth manifold, we can in addition ensure that the function *m* is smooth.

### Proof

(a) Let $\epsilon >0$ and $b_{0}\in B$. We have to prove that there exists an open neighbourhood $U_{b_{0}}$ of $b_{0}$ in *B* such that $\min (\eta )(b)>\min (\eta )(b_{0})-\epsilon$ for every $b\in U_{b_{0}}$. Suppose, on the contrary, that such a neighbourhood does not exist for some $b_{0}$. Then there exists a sequence $(b_{n},x_{n})$ of points in *K* such that $\eta (b_{n},x_{n})\le \min (\eta )(b_{0})-\epsilon$ for every $n\in \mathbb {N}$ and $\lim \limits _{n\rightarrow \infty }b_{n}=b_{0}$. The set $L=\{b_{n}\,|\,n\in \mathbb {Z}_{+}\}$ is a compact subset of *B*, hence $(\pi |_{K})^{-1}(L)$ is a compact subset of *K*. We may therefore assume, that the sequence $(b_{n},x_{n})$ is convergent with limit $(b_{0},x_{0})\in K$. But then we have

$$ \min (\eta )(b_{0})\le \eta (b_{0},x_{0})\le \min (\eta )(b_{0})-\epsilon , $$

which leads us to a contradiction.

(b) We have shown that for every $b_{0}\in B$ we can find a neighbourhood $U_{b_{0}}$ of $b_{0}$ in *B* and a number $\epsilon _{b_{0}}>0$ such that $\epsilon _{b_{0}}<\min (\eta )(b)$ for every $b\in U_{b_{0}}$. Since *B* is paracompact, we can find a subset $B'\subset B$ and for every $b\in B'$ an open subset $V_{b}\subset U_{b}$ such that $\{V_{b}\}_{b\in B'}$ is a locally finite open cover of *B*. Choose a continuous partition of unity $\{\rho _{b}\}_{b\in B'}$, subordinated to the cover $\{V_{b}\}_{b\in B'}$. The function $m(\eta ):B\rightarrow (0,\infty )$, defined by

$$ m(\eta )=\sum _{b\in B'}\epsilon _{b}\rho _{b} $$

is then continuous and satisfies $0<m(\eta )(b)<\min (\eta )(b)$ for every $b\in B$. $\square$

Classical versions of Runge and Mergelyan approximation theorems show us that we can approximate a holomorphic function on a Runge compact set *K* arbitrarily closely on *K* with global holomorphic functions on *X*. In the construction of families of proper holomorphic maps, we use the following Mergelyan theorem for proper families of compact Runge sets (see Corollary 5.2 and Remark 5.7 in [7]).

### Theorem 2.8

(Mergelyan theorem for proper families of Runge compacts) Let *B* be a paracompact Hausdorff space, $k\in \mathbb {Z}_{+}$, $0<\alpha <1$ and let $J=\{J_{b}\}_{b\in B}$ be a continuous family of complex structures on *X* of class $C^{(k,\alpha )}$, parametrised by *B*. Let $K\subset B\times X$ be a proper family of Runge compacts in *X* and let $\epsilon :B\rightarrow (0,\infty )$ be a continuous function. Then for every $f\in \mathcal {A}_{J}(K)$ there exists a function $F\in \mathcal {O}_{J}(B\times X)$ such that $\Vert F_{b}-f_{b}\Vert _{K_{b}}<\epsilon (b)$ for every $b\in B$.

In our construction, we need the following combination of the Mergelyan theorem and Proposition 2.7.

### Proposition 2.9

Let *B* be a metrisable space, $k\in \mathbb {Z}_{+}$, $0<\alpha <1$ and let $J=\{J_{b}\}_{b\in B}$ be a continuous family of complex structures on *X* of class $C^{(k,\alpha )}$, parametrised by *B*. Let $n\in \mathbb {N}$ and let $K_{1},K_{2},\ldots ,K_{n}\subset B\times X$ be wide, proper families of compact subsets of *X* such that their union is contained in a proper family *K* of Runge compacts in *X*. Suppose $f\in \mathcal {A}_{J}(K)$ is a function that satisfies conditions $\Re f>C_{i}$ on $K_{i}$ for some positive constants $C_{i}$ for $1\le i\le n$. Then for every continuous function $\epsilon :B\rightarrow (0,\infty )$ there exists a function $F\in \mathcal {O}_{J}(B\times X)$ such that $\Re F>C_{i}$ on $K_{i}$ for $1\le i\le n$ and $\Vert F_{b}-f_{b}\Vert _{K_{b}}<\epsilon (b)$ for every $b\in B$.

### Proof

For $1\le i\le n$ the function $\Re f-C_{i}$ is continuous and positive on $K_{i}$. By Proposition 2.7, there exist continuous functions $\delta _{i}:B\rightarrow (0,\infty )$ such that for every $(b,x)\in K_{i}$ we have $\Re f(b,x)-\delta _{i}(b)>C_{i}$. Now define a continuous function $\delta =\min \{\delta _{1},\delta _{2},\ldots ,\delta _{n},\epsilon \}:B\rightarrow (0,\infty )$. From Mergelyan’s theorem, it follows that there exists a function $F\in \mathcal {O}_{J}(B\times X)$ such that $\Vert F_{b}-f_{b}\Vert _{K_{b}}<\delta (b)$ for every $b\in B$. This function *F* satisfies the conditions. $\square$

## Construction of families of proper holomorphic maps

We first recall how we can reduce the construction of a family of proper holomorphic maps to the construction of a converging sequence on an exhausting family of compact sets in *X*.

### Proposition 3.1

Let *B* be a topological space, $k\in \mathbb {Z}_{+}$, $0<\alpha <1$ and let $J=\{J_{b}\}_{b\in B}$ be a continuous family of complex structures on *X* of class $C^{(k,\alpha )}$, parametrised by *B*. Let $\emptyset =K_{0}\subset K_{1}\subset K_{2}\subset K_{3}\subset \ldots$ be an exhaustion of *X* by compact sets such that $K_{n}\subset \text {Int}\,K_{n+1}$ for every $n\in \mathbb {N}$. Suppose that for every $n\in \mathbb {Z}_{+}$ we have functions $F_{n,1},F_{n,2}\in \mathcal {A}_{J}(B\times K_{n})$, such that for every $n\in \mathbb {N}$ it holds:

$(a)_{n}$: $|F_{n,i}(b,x)-F_{n-1,i}(b,x)|<\frac{1}{2^{n-1}}$ for every $(b,x)\in {B\times K_{n-1}}$ and $i=1,2$,
$$(b)_{n}$$: $$\max \{\Re F_{n,1}(b,x),\Re F_{n,2}(b,x)\}>n-1$$ for every $$(b,x)\in B\times (K_{n}\setminus \text {Int}\,K_{n-1})$$.

Then there exist functions $F_{1},F_{2}\in \mathcal {O}_{J}(B\times X)$ such that $F=(F_{1},F_{2}):B\times X\rightarrow \mathbb {C}^{2}$ is a continuous *J*-holomorphic map, for which $F_{b}:X\rightarrow \mathbb {C}^{2}$ is a proper map for every $b\in B$.

### Proof

It follows from the condition $(a)_{n}$ that the sequences $(F_{n,1})_{n\in \mathbb {N}}$ and $(F_{n,2})_{n\in \mathbb {N}}$ converge uniformly on the sets of the form $B\times K$, where $K\subset X$ is a compact subset. For the limit functions $F_{1}=\lim \limits _{n\rightarrow \infty }F_{n,1}$ and $F_{2}=\lim \limits _{n\rightarrow \infty }F_{n,2}$, we have that $F_{1},F_{2}\in \mathcal {O}_{J}(B\times X)$.

For $n>1$ and $i=1,2$ it then follows from $(a)_{n}$ that

$$ |F_{i}(b,x)-F_{n,i}(b,x)|<\frac{1}{2^{n}}+\frac{1}{2^{n+1}}+\ldots =\frac{1}{2^{n-1}}<1 \text { for } (b,x)\in {B\times K_{n}} $$

and further from $(b)_{n}$ that

$$ \max \{\Re F_{1}(b,x),\Re F_{2}(b,x)\}>n-2 \text { for } (b,x)\in B\times (K_{n}\setminus \text {Int}\,K_{n-1}), $$

which implies that $F_{b}:X\rightarrow \mathbb {C}^{2}$ is a proper map for every $b\in B$. $\square$

We now consider the case $X=\mathbb {R}^2$, where the topology is trivial:

### Theorem 3.2

Let *B* be a metrisable topological space, $k\in \mathbb {Z}_{+}$, $0<\alpha <1$ and let $J=\{J_{b}\}_{b\in B}$ be a continuous family of complex structures on $\mathbb {R}^{2}$ of class $C^{(k,\alpha )}$, parametrised by *B*. Then there exists a *J*-holomorphic map $F:B\times \mathbb {R}^2\rightarrow \mathbb {C}^{2}$ for which $F_{b}:\mathbb {R}^2\rightarrow \mathbb {C}^{2}$ is a proper map for every $b\in B$.

To prove Theorem 3.2 we first introduce some notations, where we identify $\mathbb {R}^2$ and $\mathbb {C}$ for convenience. We choose the exhaustion of $\mathbb {C}$ by closed disks

$$ K_{n}=n\overline{\mathbb {D}}=\{z\in \mathbb {C}\,:\,|z|\le n\} $$

for $n\in \mathbb {N}$, and denote by

$$ A_{n}=K_{n}\setminus \text {Int}\,K_{n-1} $$

the closed annulus in $\mathbb {C}$ between circles of radii $n-1$ and *n*. Also let $K_{0}=\emptyset$.

### Definition 3.3

(1) Let $n\in \mathbb {N}$ and suppose we have $k\in \mathbb {N}$ angles $0\le \phi _{1}<\ldots<\phi _{k}<2\pi$. We then define the following subsets of $\mathbb {C}$:

$$\begin{aligned} \gamma (n,\{\phi _{1},\ldots ,\phi _{k}\})= &   \{re^{i\phi }\,:\,r\in [n,n+1],\phi \in \{\phi _{1},\ldots ,\phi _{k}\}\}, \\ p(n,\{\phi _{1},\ldots ,\phi _{k}\})= &   \{ne^{i\phi }\,:\,\phi \in \{\phi _{1},\ldots ,\phi _{k}\}\}. \end{aligned}$$

The set $\gamma (n,\{\phi _{1},\ldots ,\phi _{k}\})$ is the union of radial line segments at angles in $\{\phi _{1},\ldots ,\phi _{k}\}$, while their inner endpoints form the set $p(n,\{\phi _{1},\ldots ,\phi _{k}\})$.

(2) Let $n\in \mathbb {N}$ and suppose $\phi _{1},\phi _{2}\in [0,2\pi ]$ are such that $0<\phi _{2}-\phi _{1}<2\pi$. We then define:

$$\begin{aligned} D(n,\phi _{1},\phi _{2})= &   \{re^{i\phi }\,:\,r\in [n,n+1],\phi \in [\phi _{1},\phi _{2}]\}, \\ \alpha (n,\phi _{1},\phi _{2})= &   \{ne^{i\phi }\,:\,\phi \in [\phi _{1},\phi _{2}]\}. \end{aligned}$$

The set $D(n,\phi _{1},\phi _{2})\subset \mathbb {C}$ is the part of the annulus $A_{n}$ which lies between angles $\phi _{1}$ and $\phi _{2}$. Its inner boundary arc is denoted by $\alpha (n,\phi _{1},\phi _{2})$.

(3) Let $n\in \mathbb {N}$, let $\phi _{1},\phi _{2}\in [0,2\pi ]$ be such that $0<\phi _{2}-\phi _{1}<2\pi$ and suppose that $0<\delta <\frac{1}{3}\min \{1,\phi _{2}-\phi _{1}\}$. We then define:

$$\begin{aligned} L(n,\delta ,\phi _{1},\phi _{2})= &   \{re^{i\phi }\,:\,r\in [n+\delta ,n+1],\phi \in [\phi _{1}+\delta ,\phi _{2}-\delta ]\}, \\ W(n,\delta ,\phi _{1},\phi _{2})= &   \overline{D(n,\phi _{1},\phi _{2})\setminus L(n,\delta ,\phi _{1},\phi _{2})}. \end{aligned}$$

The set $W(n,\delta ,\phi _{1},\phi _{2})$ is the closed $\delta$-neighbourhood of $\gamma (n,\{\phi _{1},\phi _{2}\})\cup \alpha (n,\phi _{1},\phi _{2})$ in $D(n,\phi _{1},\phi _{2})$.

(4) Let $n,k\in \mathbb {N}$ and suppose $0=\phi _{1}<\phi _{2}<\ldots<\phi _{k}<2\pi$. Furthermore, let $0<\delta <\frac{1}{3}\min \{1,\phi _{2}-\phi _{1},\phi _{3}-\phi _{2},\ldots ,\phi _{k}-\phi _{k-1},2\pi -\phi _{k}\}$. We then define:

$$\begin{aligned} W(n,\delta ,\{\phi _{1},\phi _{2},\ldots ,\phi _{k}\})= &   W(n,\delta ,\phi _{1},\phi _{2})\cup \ldots \cup W(n,\delta ,\phi _{k-1},\phi _{k})\cup W(n,\delta ,\phi _{k},2\pi ), \\ L(n,\delta ,\{\phi _{1},\phi _{2},\ldots ,\phi _{k}\})= &   L(n,\delta ,\phi _{1},\phi _{2})\cup \ldots \cup L(n,\delta ,\phi _{k-1},\phi _{k})\cup L(n,\delta ,\phi _{k},2\pi ). \end{aligned}$$

(5) Let $n\in \mathbb {N}$ and suppose $0=\phi _{1}<\phi _{2}<\ldots<\phi _{k}<2\pi$, where *k* is an even number. We then define:

$$\begin{aligned} D_{\text {odd}}(n,\{\phi _{1},\phi _{2},\ldots ,\phi _{k}\})= &   D(n,\phi _{1},\phi _{2})\cup D(n,\phi _{3},\phi _{4})\cup \ldots \cup D(n,\phi _{k-1},\phi _{k}), \\ D_{\text {even}}(n,\{\phi _{1},\phi _{2},\ldots ,\phi _{k}\})= &   D(n,\phi _{2},\phi _{3})\cup D(n,\phi _{4},\phi _{5})\cup \ldots \cup D(n,\phi _{k},2\pi ). \end{aligned}$$

In a similar fashion we define the sets $\alpha _{\text {odd}}$, $\alpha _{\text {even}}$, $L_{\text {odd}}$, $L_{\text {even}}$, $W_{\text {odd}}$ and $W_{\text {even}}$.

**Fig. 1:**
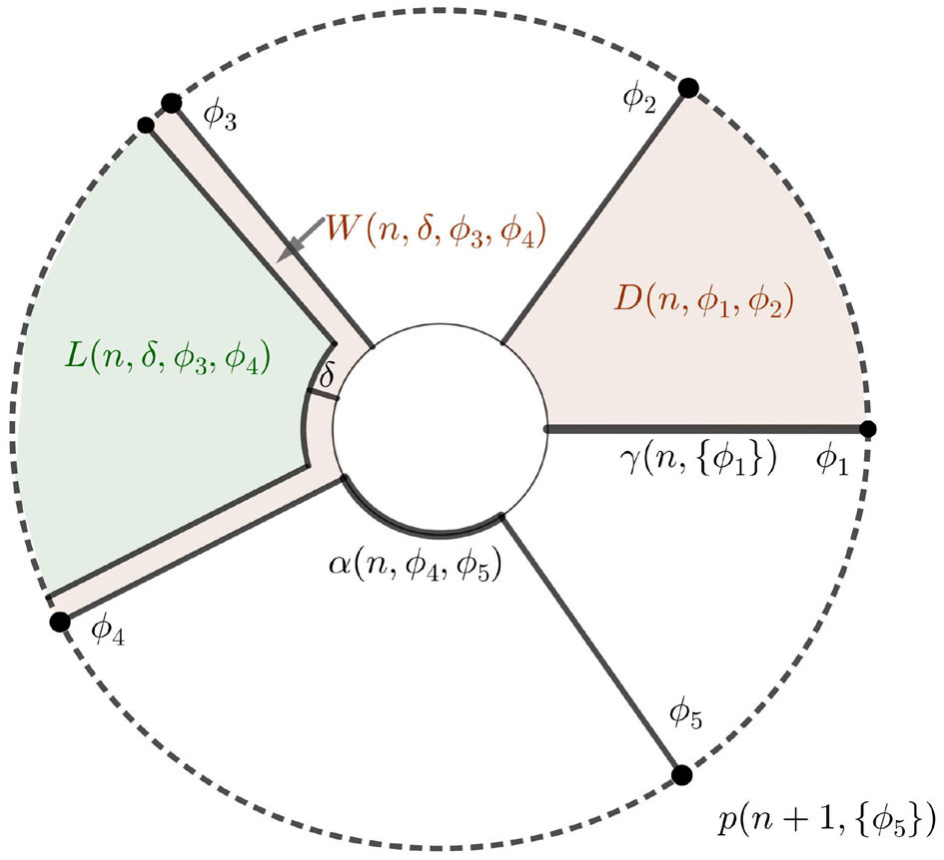
Pictures of sets from Definition 3.3

All of the above sets are compact subsets of $\mathbb {C}$ as shown in Figure 1.

Next we extend the above definitions to the setting of $B\times \mathbb {C}$, where *B* is a topological space. If $\phi _{i}:B\rightarrow [0,2\pi ]$ and $\delta :B\rightarrow (0,\frac{1}{3})$ are continuous functions that satisfy the conditions $(1)-(5)$ in Definition 3.3 pointwise, we can define families of compact subsets of $\mathbb {C}$ whose fibres are the corresponding sets. We denote such a family by adding a subscript *B*. For example, if $n\in \mathbb {N}$ and $\phi _{1},\phi _{2}:B\rightarrow [0,2\pi ]$ are continuous functions such that $0<\phi _{2}(b)-\phi _{1}(b)<2\pi$ for every $b\in B$, then $D_{B}(n,\phi _{1},\phi _{2})$ is a subset of $B\times \mathbb {C}$, which is implicitly defined by

$$ (D_{B}(n,\phi _{1},\phi _{2}))_{b}=D(n,\phi _{1}(b),\phi _{2}(b))\subset \mathbb {C}$$

for every $$b\in B$$.

### Proposition 3.4

Let *B* be a topological space. The sets $\gamma _{B}, p_{B}, D_{B},\alpha _{B},L_{B}$ and $W_{B}$ are all proper families of compact subsets of $\mathbb {C}$. The same is true for their odd and even versions.

### Proof

All these sets are subsets of the constant family $B\times K_{n+1}$, so by Proposition 2.6 it suffices to prove they are closed subsets. This follows from the fact that their complements in $B\times \mathbb {C}$ are open since the functions $\phi _{i}$ and $\delta$ are continuous. $\square$

To be able to use the Mergelyan theorem to construct the sequence of functions from Proposition 3.1, we need the following result.

### Proposition 3.5

Let *B* be a metrisable topological space, $n\in \mathbb {N}$ and let $\phi _{1},\phi _{2}:B\rightarrow [0,2\pi ]$ be continuous functions such that $0<\phi _{2}(b)-\phi _{1}(b)<2\pi$ for every $b\in B$. Suppose $f:D_{B}(n,\phi _{1},\phi _{2})\rightarrow \mathbb {C}$ is a continuous function such that:

1. $$\Re f>n$$ on $$\gamma _{B}(n,\{\phi _{1},\phi _{2}\})\cup \alpha _{B}(n,\phi _{1},\phi _{2})$$,
2. $$\Re f>n+1$$ on $$p_{B}(n+1,\{\phi _{1},\phi _{2}\})$$.

Then there exists a continuous function $\delta :B\rightarrow (0,\frac{1}{3})$, satisfying $\delta <\tfrac{1}{3}\min \{1,\phi _{2}-\phi _{1}\}$ and such that:

1. $$\Re f>n$$ on $$W_{B}(n,\delta ,\phi _{1},\phi _{2})$$,
2. $$\Re f>n+1$$ on $$\alpha _{B}(n+1,\phi _{1},\phi _{1}+\delta )\cup \alpha _{B}(n+1,\phi _{2}-\delta ,\phi _{2})$$.

### Proof

Let us define

$$\begin{aligned} K'=&\{(b,x)\in D_{B}(n,\phi _{1},\phi _{2}):\Re f(b,x)\le n\}\cup \{(b,x)\in \alpha _{B}(n+1,\phi _{1},\phi _{2})\\&:\Re f(b,x)\le n+1)\}. \end{aligned}$$

The set $K'$ is a closed subset of the proper family of compact subsets $D_{B}(n,\phi _{1},\phi _{2})$, hence $K'$ is a proper family as well. Since $K'$ may have empty fibers, we enlarge it to a proper family of compact subsets

$$ K=K'\cup p_{B}(n+1,\{\tfrac{1}{2}(\phi _{1}+\phi _{2})\}), $$

for which $K_{b}\ne \emptyset$ for every $b\in B$. Then *K* is a wide, proper family of compact subsets of $\mathbb {C}$ which is disjoint from $\gamma _{B}(n,\{\phi _{1},\phi _{2}\})\cup \alpha _{B}(n,\phi _{1},\phi _{2})$.

For any $(b,x)\in K$ we can write *x* in the form $x=re^{i\phi }$ for unique $r\in (n,n+1]$ and $\phi \in (\phi _{1}(b),\phi _{2}(b))$. The functions $\phi _{2}-\phi$, $\phi -\phi _{1}$ and $r-n$ are continuous and positive on *K*. By Proposition 2.7, we can find a function $\delta :B\rightarrow (0,\infty )$ such that for every $(b,re^{i\phi })\in K$ we have:

$$\begin{aligned} r\in &   (n+\delta (b),n+1], \\ \phi\in &   (\phi _{1}(b)+\delta (b),\phi _{2}(b)-\delta (b)). \end{aligned}$$

If needed, we can make $\delta$ smaller, so that $\delta <\tfrac{1}{3}\min \{1,\phi _{2}-\phi _{1}\}$. We then have $\Re f>n$ on $W_{B}(n,\delta ,\phi _{1},\phi _{2})$ and $\Re f>n+1$ on $\alpha _{B}(n+1,\phi _{1},\phi _{1}+\delta )\cup \alpha _{B}(n+1,\phi _{2}-\delta ,\phi _{2})$. $\square$

### Proof of Theorem 3.2

Let $l_{n}=3^{n-1}$ for $n\in \mathbb {N}$. According to Proposition 3.1, it suffices to construct a sequence of functions $F_{n,1},F_{n,2}\in \mathcal {A}_{J}(B\times K_{n})$ that for every $n\in \mathbb {N}$ satisfy the conditions:

$(a)_{n}$: $|F_{n,i}(b,x)-F_{n-1,i}(b,x)|<\frac{1}{2^{n-1}}$ for every $(b,x)\in {B\times K_{n-1}}$ and $i=1,2$,
$$(b)_{n}$$: $$\max \{\Re F_{n,1}(b,x),\Re F_{n,2}(b,x)\}>n-1$$ for every $$(b,x)\in B\times A_{n}$$.

We construct such a sequence inductively, together with the sequence of continuous families of angles: These angles are defined by continuous functions $\phi _{n,j}:B\rightarrow [0,2\pi ]$ for $j\in \{1,\ldots ,2l_{n}+1\}$, which satisfy for every $n\in \mathbb {N}$ the following conditions:

$$(c)_{n}$$: $$0=\phi _{n,1}(b)<\phi _{n,2}(b)<\cdots<\phi _{n,2l_{n}}(b)<2\pi =\phi _{n,2l_{n}+1}(b)$$ for every $$b\in B$$,
$$(d)_{n}$$: $$\Re F_{n,1}>n$$ on $$(\alpha _{\text {odd}})_{B}(n,\{\phi _{n,1},\phi _{n,2},\ldots ,\phi _{n,2l_{n}}\})$$, $$\Re F_{n,2}>n$$ on $$(\alpha _{\text {even}})_{B}(n,\{\phi _{n,1},\phi _{n,2},\ldots ,\phi _{n,2l_{n}}\})$$.

To start with the induction, we define constant functions $\phi _{1,1},\phi _{1,2},\phi _{1,3}:B\rightarrow [0,2\pi ]$ by

$$ \phi _{1,1}=0,\,\phi _{1,2}=\pi ,\,\phi _{1,3}=2\pi $$

and choose any functions $F_{0,1},F_{0,2}\in \mathcal {A}_{J}(B\times K_{0})$ and $F_{1,1},F_{1,2}\in \mathcal {A}_{J}(B\times K_{1})$ that satisfy the conditions $(a)_1$, $(b)_{1}$ and $(d)_{1}$. (For example, we could just choose appropriate constant functions $F_{0,1}$, $F_{0,2}$, $F_{1,1}$ and $F_{1,2}$.)

Suppose that for some $n\in \mathbb {N}$ we have functions $F_{m,1},F_{m,2}\in \mathcal {A}_{J}(B\times K_{m})$ and $\phi _{m,j}:B\rightarrow [0,2\pi ]$ for $j\in \{1,2,\ldots ,2l_{m}+1\}$ which satisfy conditions $(a)_{m}$, $(b)_{m}$, $(c)_{m}$ and $(d)_{m}$ for $m\in \{1,2,\ldots ,n\}$. In the induction step, we construct continuous functions

$$\phi _{n+1,1},\phi _{n+1,2},\ldots ,\phi _{n+1,2l_{n+1}+1}:B\rightarrow [0,2\pi ],$$

that satisfy

$$ 0=\phi _{n+1,1}<\phi _{n+1,2}<\ldots<\phi _{n+1,2l_{n+1}}<2\pi =\phi _{n+1,2l_{n+1}+1} $$

and functions $F_{n+1,1},F_{n+1,2}\in \mathcal {A}_{J}(B\times K_{n+1})$ that satisfy $(a)_{n+1}$, $(b)_{n+1}$ and $(d)_{n+1}$. Before we turn to details let us quickly describe the main idea of the induction step. We need to construct functions $F_{n+1,1},F_{n+1,2}\in \mathcal {A}_{J}(B\times K_{n+1})$ for which $\max \{\Re F_{n+1,1},\Re F_{n+1,2}\}>n$ on $B\times A_{n+1}$. To do that, we split the inductive step into three parts. In the first part, we use Mergelyan’s theorem to construct functions $\tilde{F}_{n,1},\tilde{F}_{n,2}\in \mathcal {O}_{J}(B\times \mathbb {R}^{2})$ which satisfy $\max \{\Re \tilde{F}_{n,1},\Re \tilde{F}_{n,2}\}>n$ on the subset $\gamma _{B}(n,\{\phi _{n,1},\phi _{n,2},\ldots ,\phi _{n,2l_{n}}\})$ of $B\times A_{n+1}$. Next we use Proposition 3.5 to show that there exists a continuous function $\delta :B\rightarrow (0,\frac{1}{3})$ such that $\max \{\Re \tilde{F}_{n,1},\Re \tilde{F}_{n,2}\}>n$ on the subset $W_{B}(n,\{\phi _{n,1},\phi _{n,2},\ldots ,\phi _{n,2l_{n}}\})$ of $B\times A_{n+1}$. In the third part, we use the idea from the proofs in [1, 3] to obtain functions $F_{n+1,1},F_{n+1,2}\in \mathcal {A}_{J}(B\times K_{n+1})$ for which $\max \{\Re F_{n+1,1},\Re F_{n+1,2}\}>n$ on $B\times A_{n+1}$.

Let us now describe the details. First we construct functions $\tilde{F}_{n,1},\tilde{F}_{n,2}\in \mathcal {O}_{J}(B\times \mathbb {R}^{2})$ that satisfy:

$$(a^{1})_{n+1}$$: $$|\tilde{F}_{n,i}(b,x)-F_{n,i}(b,x)|<\frac{1}{2^{n+1}}$$ for $$(b,x)\in {B\times K_{n}}$$ and $$i=1,2$$,
$$(b^{1})_{n+1}$$: $$\Re \tilde{F}_{n,1}>n$$ on $$\gamma _{B}(n,\{\phi _{n,1},\ldots ,\phi _{n,2l_{n}}\})\cup (\alpha _{\text {odd}})_{B}(n,\{\phi _{n,1},\ldots ,\phi _{n,2l_{n}}\})$$, $$\Re \tilde{F}_{n,2}>n$$ on $$\gamma _{B}(n,\{\phi _{n,1},\ldots ,\phi _{n,2l_{n}}\})\cup (\alpha _{\text {even}})_{B}(n,\{\phi _{n,1},\ldots ,\phi _{n,2l_{n}}\})$$,
$$(d^{1})_{n+1}$$: $$\Re \tilde{F}_{n,i}>n+1$$ on $$p_{B}(n+1,\{\phi _{n,1},\ldots ,\phi _{n,2l_{n}}\})$$ for $$i=1,2$$.

To do that we first continuously extend the functions $F_{n,1},F_{n,2}$ from the set $B\times K_{n}$ to the set $(B\times K_{n})\cup \gamma _{B}(n,\{\phi _{n,1},\ldots ,\phi _{n,2l_{n}}\})$ so that $\Re F_{n,i}>n$ on $\gamma _{B}(n,\{\phi _{n,1},\ldots ,\phi _{n,2l_{n}}\})$ and $\Re F_{n,i}>n+1$ on $p_{B}(n+1,\{\phi _{n,1},\ldots ,\phi _{n,2l_{n}}\})$ for $i=1,2$. Now note that $\Re F_{n,1}>n$ on the proper family $\gamma _{B}(n,\{\phi _{n,1},\ldots ,\phi _{n,2l_{n}}\})\cup (\alpha _{\text {odd}})_{B}(n,\{\phi _{n,1},\ldots ,\phi _{n,2l_{n}}\})$ and that $\Re F_{n,1}>n+1$ on the proper family $p_{B}(n+1,\{\phi _{n,1},\ldots ,\phi _{n,2l_{n}}\})$ of compact subsets of $\mathbb {R}^{2}$. The union of these two proper families is contained in the proper family $(B\times K_{n})\cup \gamma _{B}(n,\{\phi _{n,1},\ldots ,\phi _{n,2l_{n}}\})$ of Runge compacts in $\mathbb {R}^{2}$, so by Proposition 2.9 we can find a function $\tilde{F}_{n,1}\in \mathcal {O}_{J}(B\times \mathbb {R}^{2})$ that satisfies $(a^{1})_{n+1}$, $(b^{1})_{n+1}$ and $(d^{1})_{n+1}$. In a similar fashion we also obtain a function $\tilde{F}_{n,2}\in \mathcal {O}_{J}(B\times \mathbb {R}^{2})$ which approximates the function $F_{n,2}$.

We now proceed to the second part of the induction step. Consider the function $\tilde{F}_{n,1}$ on the proper family $(D_{\text {odd}})_{B}(n,\{\phi _{n,1},\ldots ,\phi _{n,2l_{n}}\})$ of compact subsets of $\mathbb {R}^{2}$. From Proposition 3.5 it follows that there exists a continuous function $\delta _{1}:B\rightarrow (0,\frac{1}{3})$ such that:

$$\cdot $$: $$\Re \tilde{F}_{n,1}>n$$ on $$(W_{\text {odd}})_{B}(n,\delta _{1},\{\phi _{n,1},\ldots ,\phi _{n,2l_{n}}\})$$,
$$\cdot $$: $$\Re \tilde{F}_{n,1}>n+1$$ on $$\bigcup \limits _{k=1}^{l_{n}}\left( \alpha _{B}(n+1,\phi _{n,2k-1},\phi _{n,2k-1}+\delta _{1})\cup \alpha _{B}(n+1, \right. $$ $$ \left. \phi _{n,2k}-\delta _{1},\phi _{n,2k})\right) $$.

In the sequel, we repeat this argument for the function $\tilde{F}_{n,2}$ on the $(D_{\text {even}})_{B}(n,\{\phi _{n,1},\ldots ,$ $\phi _{n,2l_{n}}\})$ to obtain a function $\delta _{2}:B\rightarrow (0,\frac{1}{3})$ such that $\tilde{F}_{n,2}$ satisfies conditions:

$$\cdot $$: $$\Re \tilde{F}_{n,2}>n$$ on $$(W_{\text {even}})_{B}(n,\delta _{2},\{\phi _{n,1},\ldots ,\phi _{n,2l_{n}}\})$$,
$$\cdot $$: $$\Re \tilde{F}_{n,2}>n+1$$ on $$\bigcup \limits _{k=1}^{l_{n}}\left( \alpha _{B}(n+1,\phi _{n,2k},\phi _{n,2k}+\delta _{2})\cup \alpha _{B}(n+1,\phi _{n,2k+1}\right. $$ $$ \left. -\delta _{2},\phi _{n,2k+1})\right) $$.

Let $\delta =\min \{\delta _{1},\delta _{2}\}$ and define functions $\phi _{n+1,1},\phi _{n+1,2},\ldots ,\phi _{n+1,2l_{n+1}+1}:B\rightarrow [0,2\pi ]$ by:

$$\begin{aligned} \phi _{n+1,3k+1}= &   \phi _{n,k+1}, \\ \phi _{n+1,3k+2}= &   \phi _{n,k+1}+\delta , \\ \phi _{n+1,3k+3}= &   \phi _{n,k+2}-\delta \end{aligned}$$

for $k\in \{0,1,\ldots ,2l_{n}-1\}$ and $\phi _{n+1,2l_{n+1}+1}=2\pi$. Observe that these functions satisfy the condition $(c)_{n+1}$ while functions $\tilde{F}_{n,1},\tilde{F}_{n,2}$ satisfy the conditions:

$(a^{2})_{n+1}$: $|\tilde{F}_{n,i}(b,x)-F_{n,i}(b,x)|<\frac{1}{2^{n+1}}$ for all $(b,x)\in {B\times K_{n}}$ and $i=1,2$,
$$(b^{2})_{n+1}$$: $$\max \{\Re \tilde{F}_{n,1}(b,x),\Re \tilde{F}_{n,2}(b,x)\}>n$$ for all $$(b,x)\in W_{B}(n,\delta ,\{\phi _{n,1},\phi _{n,2},\ldots ,$$ $$\phi _{n,2l_{n}}\})$$,
$$(d^{2})_{n+1}$$: $$\Re \tilde{F}_{n,1}>n+1$$ on $$\bigcup \limits _{k=1}^{l_{n}}\left( \alpha _{B}(n+1,\phi _{n,2k-1},\phi _{n,2k-1}+\delta )\cup \alpha _{B}(n+1,\phi _{n,2k}\right. $$ $$\left. -\delta ,\phi _{n,2k})\right) $$, $$\Re \tilde{F}_{n,2}>n+1$$ on $$\bigcup \limits _{k=1}^{l_{n}}\left( \alpha _{B}(n+1,\phi _{n,2k},\phi _{n,2k}+\delta )\cup \alpha _{B}(n+1,\phi _{n,2k+1}\right. $$ $$\left. -\delta ,\phi _{n,2k+1})\right) $$.

**Fig. 2:**
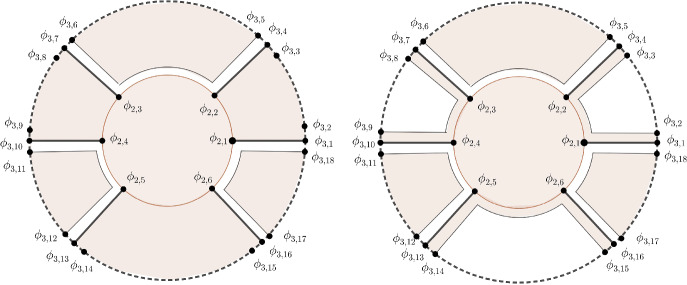
Regions in the inductive step in the case $n=2$

In the third part of the induction step, we correct the functions $\tilde{F}_{n,1},\tilde{F}_{n,2}$ so that we obtain the condition $(b)_{n+1}$ on the set $L_{B}(n,\delta ,\{\phi _{n,1},\ldots ,\phi _{n,2l_{n}}\})$ as well as the condition $(d)_{n+1}$ on the remaining arcs. Let us define a proper family of Runge compacts by

$$ (A_{\text {odd}})_{n}{=}(B\times K_{n})\cup (D_{\text {odd}})_{B}(n,\{\phi _{n,1},\ldots ,\phi _{n,2l_{n}}\})\cup (L_{\text {even}})_{B}(n,\delta ,\{\phi _{n,1},\ldots ,\phi _{n,2l_{n}}\}), $$

see the left part of Figure 2, and define the function $\overline{F}_{n,1}\in \mathcal {A}_{J}((A_{\text {odd}})_{n})$ by

$$ \overline{F}_{n,1}(b,x)= \left\{ \begin{array} {ll} \tilde{F}_{n,1} & ;(b,x)\in (B\times K_{n})\cup (D_{\text {odd}})_{B}(n,\{\phi _{n,1},\ldots ,\phi _{n,2l_{n}}\}),\\ n+2 & ;\hspace{1mm}(b,x)\in (L_{\text {even}})_{B}(n,\delta ,\{\phi _{n,1},\ldots ,\phi _{n,2l_{n}}\}). \end{array}\right. $$

Function $\overline{F}_{n,1}$ satisfies conditions:

$$\cdot $$: $$|\overline{F}_{n,1}(b,x)-F_{n,1}(b,x)|<\frac{1}{2^{n+1}}$$ for $$(b,x)\in {B\times K_{n}}$$,
$$\cdot $$: $$\Re \overline{F}_{n,1}>n$$ on $$(W_{\text {odd}})_{B}(n,\delta ,\{\phi _{n,1},\ldots ,\phi _{n,2l_{n}}\}) \cup (L_{\text {even}})_{B}(n,\delta ,\{\phi _{n,1},\ldots ,\phi _{n,2l_{n}}\})$$,
$$\cdot $$: $$\Re \overline{F}_{n,1}>n+1$$ on $$(\alpha _{\text {odd}})_{B}(n+1,\{\phi _{n+1,1},\ldots ,\phi _{n+1,2l_{n+1}}\})$$.

By applying Proposition 2.9 to the function $\overline{F}_{n,1}$ with precision at least $\frac{1}{2^{n+1}}$ we obtain a function $F_{n+1,1}\in \mathcal {O}_{J}(B\times \mathbb {R}^{2})$ that satisfies:

$$(a^{3,1})_{n+1}$$: $$|F_{n+1,1}(b,x)-F_{n,1}(b,x)|<\frac{1}{2^{n}}$$ for $$(b,x)\in {B\times K_{n}}$$,
$$(b^{3,1})_{n+1}$$: $$\Re F_{n+1,1}>n$$ on $$(W_{\text {odd}})_{B}(n,\delta ,\{\phi _{n,1},\ldots ,\phi _{n,2l_{n}}\}) \cup (L_{\text {even}})_{B}$$ $$(n,\delta ,\{\phi _{n,1},\ldots ,\phi _{n,2l_{n}}\})$$,
$$(d^{3,1})_{n+1}$$: $$\Re F_{n+1,1}>n+1$$ on $$(\alpha _{\text {odd}})_{B}(n+1,\{\phi _{n+1,1},\ldots ,\phi _{n+1,2l_{n+1}}\})$$.

Similarly we obtain a function $F_{n+1,2}\in \mathcal {O}_{J}(B\times \mathbb {R}^{2})$ which satisfies conditions:

$$(a^{3,2})_{n+1}$$: $$|F_{n+1,2}(b,x)-F_{n,2}(b,x)|<\frac{1}{2^{n}}$$ for $$(b,x)\in {B\times K_{n}}$$,
$$(b^{3,2})_{n+1}$$: $$\Re F_{n+1,2}>n$$ on $$(W_{\text {even}})_{B}(n,\delta ,\{\phi _{n,1},\ldots ,\phi _{n,2l_{n}}\}) \cup (L_{\text {odd}})_{B}$$ $$(n,\delta ,\{\phi _{n,1},\ldots ,\phi _{n,2l_{n}}\})$$,
$$(d^{3,2})_{n+1}$$: $$\Re F_{n+1,2}>n+1$$ on $$(\alpha _{\text {even}})_{B}(n+1,\{\phi _{n+1,1},\ldots ,\phi _{n+1,2l_{n+1}}\})$$.

The areas in $B\times A_{n+1}$ where $\Re F_{n+1,1}>n$ respectively $\Re F_{n+1,2}>n$ are shown in the right part of Figure 2. Condition $(a)_{n+1}$ now follows from conditions $(a^{3,1})_{n+1}$ and $(a^{3,2})_{n+1}$, condition $(b)_{n+1}$ follows from conditions $(b^{3,1})_{n+1}$ and $(b^{3,2})_{n+1}$ while condition $(d)_{n+1}$ follows from conditions $(d^{3,1})_{n+1}$ and $(d^{3,2})_{n+1}$. The proof is concluded by applying Proposition 3.1. $\square$

### Proof of Theorem 1.1

Let $K_0=\emptyset$. Choose a point $b_0\in B$, and a strongly $J_{b_0}$-subharmonic Morse exhaustion function $\tau :X\rightarrow (0,\infty )$. By a small perturbation, we may assume that there is exactly one critical point at every critical level set. Choose an increasing sequence $(c_n)_{n\in \mathbb {N}}$ of regular values of $\tau$ converging to $\infty$ such that the interval $(c_n,c_{n+1})$ contains at most one critical value of $\tau$. Then $K_n=\{x\in X\,:\,\tau (x)\le c_n\}$ is a smoothly bounded compact Runge set, and we may assume that $c_1$ is chosen so large that $K_1$ is nonempty and so small that it is simply connected. Then $bK_{n}$ is a union of finitely many, say $k_n$, smooth closed Jordan curves. If $\tau$ has no critical values in $(c_n,c_{n+1})$, then $K_{n+1}\setminus \text {Int}\,K_n$ is a union of $k_n$ annular regions, and we call this the *noncritical case*. In this case, there is no change in the topology, and the construction is similar to the construction in the proof of Theorem 3.2. We will explain the details below. In the *critical case*, $\tau$ has exactly one critical point in $K_{n+1}\setminus \text {Int}\,K_n$ of index 0 or 1.

If its index is 0, then it is a minimum of $\tau$ and a new simply connected component appears. If its index is 1, then there is a compact Jordan arc $\gamma _n\subset \text {Int}\,K_{n+1}\setminus \text {Int}\,K_n$ transversally attached with both endpoints to $K_n$, and otherwise disjoint from $K_n$, such that $K_n \cup \gamma _n$ is a Runge set and a strong deformation retract of $K_{n+1}$. We need to distinguish two cases: either the endpoints of the arc $\gamma _n$ lie on the same component of $bK_n$ or the arc connects two different components of $bK_n$. We choose two distinct points, denoted by $p^j_n$ and $q^j_n$ on each boundary component of $bK_n$ ($j=1,\ldots , k_n$) such that the endpoints of the arc $\gamma _n$ are $p^j_n$ and $q^l_n$ for some $j,l\in \{1,\ldots , k_n\}$. The map *F* is constructed inductively and at the critical case, we need to continuously extend the maps $F_{n,1},F_{n,2}:B\times b\gamma _n\rightarrow \{z\in \mathbb {C}:\Re z>n\}$ to maps $F_{n,1},F_{n,2}:B\times \gamma _n\rightarrow \{z\in \mathbb {C}:\Re z>n\}$. This is possible since the set $\{z\in \mathbb {C}:\Re z>n\}$ is contractible. Moreover, we will also obtain a continuously varying family of points on each boundary component of $K_n$, which corresponds to the continuous family of angles in the proof of Theorem 3.2, and the points $p^j_n$ and $q^j_n$ will correspond to the constant angles with the different parity: for this reason, we choose for each *n* and for each $j\in \{1,\ldots k_n\}$ a continuous map $\varphi _n^j$ from $[0,2\pi ]$ to the *j*-th component of $bK_n$ which induces a homeomorphism from the quotient $[0,2\pi ]/(0\sim 2\pi )$ to the *j*-th component of $bK_n$, inducing the given orientation. Furthermore, we may achieve that $\varphi _{n}^{j}(0)=\varphi _{n}^{j}(2\pi )=p^{j}_{n}$ and $\varphi _{n}^{j}(\pi )=q^{j}_{n}$.

We inductively construct functions $F_{n,1},F_{n,2}\in \mathcal {A}_{J}(B\times K_{n})$, $n\in \mathbb {N}\cup \{0\}$, positive integers $l^j_{n}$, $j\in \{1,\ldots , k_n\}, n\in \mathbb {N}$, continuous functions $\phi _{n,m}^j:B\rightarrow [0,2\pi ]$, $m\in \{1, \ldots , 2l^j_{n}+1\}, j\in \{1,\ldots , k_n\}, n\in \mathbb {N}$, that satisfy the following conditions for every $n\in \mathbb {N}$:

$$(a)_n$$: $$|F_{n,i}(b,x)-F_{n-1,i}(b,x)|<\frac{1}{2^{n-1}}$$ for $$(b,x)\in {B\times K_{n-1}}$$ and $$i=1,2$$,
$$(b)_n$$: $$\max \{\Re F_{n,1}(b,x),\Re F_{n,2}(b,x)\}>n-1$$ for every $$(b,x)\in B\times (K_{n}\setminus \text {Int}\,K_{n-1})$$,
$(c)_n$: $0=\phi _{n,1}^j(b)<\phi _{n,2}^j(b)<\cdots<\phi _{n,2l^j_{n}}^j(b)<2\pi =\phi _{n,2l^j_{n}+1}^j(b)$ for each $b\in B$ and $j\in \{1,\ldots , k_n\}$; for each $j\in \{1,\ldots , k_n\}$ there is $m^j_{n}\in \{1, \ldots , l^j_{n}\}$ such that $\phi _{n,2m^j_{n}}^j\equiv \pi$,
$(d)_n$: $\Re F_{n,1}(b,x)>n$ for $x\in \varphi _n^j ([\phi _{n,2m-1}^j(b),\phi _{n,2m}^j(b)])$ and $\Re F_{n,2}(b,x)>n$ for $x\in \varphi _n^j ([\phi _{n,2m}^j(b),\phi _{n,2m+1}^j(b)])$ for each $m\in \{1, \ldots , l^j_{n}\}$, and $j\in \{1,\ldots , k_n\}$.

Once we complete the construction, the proof is complete due to Proposition 3.1.

To start the induction take $F_{0,1}=F_{0,2}=F_{1,1}=F_{1,2}=2$, $l_1^j=1$, $m_1^j=1$ for $j\in \{1,\ldots , k_1\}$, which satisfy $(a)_{1}-(d)_{1}$.

Assume we have already constructed $F_{i,1},F_{i,2}$, $l^j_{i}$, $m^j_{i}$, $\phi _{i,m}^j$, $m\in \{1, \ldots , 2l^j_{i}+1\}$, $j\in \{1,\ldots , k_i\}$, $i\in \{1,\ldots n\}$, that satisfy $(a)_{i}-(d)_{i}$ for all $i\in \{1,\ldots n\}$. We construct the functions $F_{n+1,1},F_{n+1,2}$ by dividing each component of the set $K_{n+1}\setminus \text {Int}\,K_{n}$ into two unions of simply connected regions that play the roles of $(D_{\text {odd}})_{B}$ and $(D_{\text {even}})_{B}$ in the proof of Theorem 3.2.

In the noncritical case, the set $K_{n+1}\setminus \text {Int}\,K_{n}$ is homeomorphic to a disjoint union of $k_{n}=k_{n+1}$ annular components which we denote by $A_{n}^{j}$ for $j=1,2,\ldots ,k_{n}$. Suppose that the boundary components of $A_{n}^{j}$ are parametrised by $\varphi _{n}^{j_{\text {inner}}}$ and $\varphi _{n+1}^{j_{\text {outer}}}$. We may choose a diffeomorphism $\psi ^{j}_{n}:\{z\in \mathbb {C}\,:\,1\le |z|\le 2\} \rightarrow A^{j}_{n}$ such that $\psi ^{j}_{n}(\textrm{e}^{i t}) =\varphi _{n}^{j_{\text {inner}}}(t)$ and $\psi ^{j}_{n}(2\textrm{e}^{i t})=\varphi _{n+1}^{j_{\text {outer}}}(t)$ for $t\in [0,2\pi )$. Denote by $\gamma '^{j}_{n}$ the arc $\psi ^{j}_{n}([1,2])$ and by $\gamma ''^{j}_{n}$ the arc $\psi ^{j}_{n}([-2,-1])$. By the property $(d)_n$ we can continuously extend the maps $F_{n,1},F_{n,2}$ from $B\times K_{n}$ to maps from $B\times (K_{n}\cup \gamma '^j_n\cup \gamma ''^j_n)$ so that the image of $B\times (\gamma '^j_n\cup \gamma ''^j_n)$ lies in $\{z\in \mathbb {C}:\Re z>n\}$ and the image of $B\times \{p_{n+1}^{j}\}$ and of $B\times \{q_{n+1}^{j}\}$ lies in $\{z\in \mathbb {C}:\Re z>n+1\}$. Then we proceed as in the proof of Theorem 3.2 to obtain functions $F_{n+1,1},F_{n+1,2}\in \mathcal {A}_{J}(B\times K_{n+1})$, integers $l^j_{n+1}$, $m^j_{n+1}$, and functions $\phi _{n+1,i}^j$ ($i\in \{1, \ldots , 2l^j_{n+1}+1\}$, $j\in \{1,\ldots , k_{n+1}\}$) satisfying properties $(a)_{n+1}-(d)_{n+1}$.

**Fig. 3:**
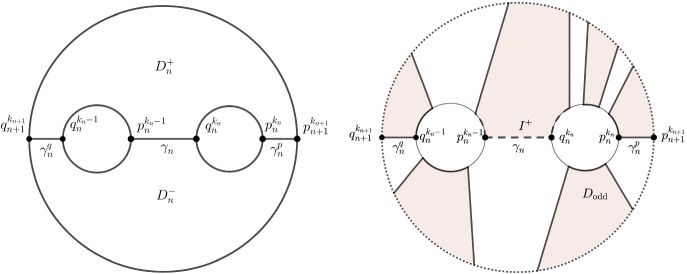
Critical case 1

In the critical case, we only need to consider critical points with the index 1, since we can treat the new appearing component in the case of critical points with index 0 in the same way as at the start of the inductive construction. Thus, we first consider the situation in which the arc $\gamma _n$ connects two different components of $bK_n$. Then the number of components of $bK_{n+1}$ is one less than the number of components of $bK_{n}$. By rearranging the notation, we may assume that $\gamma _n$ connects $p^{k_n-1}_n$ and $q^{k_n}_n$. The set $K_{n+1}\setminus \text {Int}\,K_{n}$ is a union of a two-connected domain $D_n$ in *X*, and perhaps a finite number of annuli, where the arc $\gamma _n\subset D_n$ connects two components of the complement of $D_n$ in *X*. In the annular regions of $K_{n+1}\setminus \text {Int}\,K_{n}$ we proceed as in the noncritical case, thus we provide the details only for the construction corresponding the domain $D_n$. Since the domain $D_n$ is two-connected, we first explain how we choose continuous family of arcs connecting the boundary of $bK_n$ and $bK_{n+1}$, corresponding to the arcs $\gamma (n,\{\phi _1,\ldots ,\phi _k\})$ in the proof of Theorem 3.2. We can choose pairwise disjoint smooth arcs $\gamma ^{p}_n$ and $\gamma ^{q}_n$ in $K_{n+1}\setminus (\text {Int}\,K_n \cup \gamma _n)$ which intersect $bK_n$ and $bK_{n+1}$ transversally at their endpoints such that the endpoints of $\gamma ^{p}_n$ are $p_n^{k_n}$ and $p_{n+1}^{k_{n+1}}$, and the endpoints of $\gamma ^{q}_n$ are $q_{n}^{k_n-1}$ and $q_{n+1}^{k_{n+1}}$, see the left part of Figure 3.

Then the domain $$D_{n}$$ is the union of two closed simply connected domains $$D_{n}^{+}$$ and $$D_{n}^{-}$$ with the arcs $$\gamma _{n}$$, $$\gamma ^{p}_{n}$$ and $$\gamma ^{q}_{n}$$ as their common boundary. There is a diffeomorphism $$\Psi _{n}^{+}$$ from $$D_{n}^{+}$$ to the convex hull *C* of points $$(2,0), (2,1), (1,2), (-1,2), (-2,1),$$ $$ (-2,0)$$ in $$\mathbb {R}^{2}$$ that maps $$q_{n+1}^{k_{n+1}}$$ to $$(-2,0)$$, $$q_{n}^{k_{n}-1}$$ to $$(-2,1)$$, $$p_{n}^{k_{n}-1}$$ to $$(-1,2)$$, $$q_{n}^{k_{n}}$$ to (1, 2), $$p_{n}^{k_{n}}$$ to (2, 1), and $$p_{n+1}^{k_{n+1}}$$ to (2, 0) (and similarly for $$D_{n}^{-}$$). The vertical segments in *C* provide arcs in $$D_{n}^{+}$$. More precisely, for any $$\phi \in (0,\pi )$$ the points $$\varphi _{n}^{k_{n-1}}(\phi )$$, $$\varphi _n^{k_{n}}(\phi )$$ from $$bK_{n}$$ are mapped to points $$(x',y')$$, $$(x'',y'')$$ for some $$x'\in (-2,-1)$$, $$x''\in (1,2)$$ and $$y',y''>0$$ on the beveled edges of *C*. Then segments from $$(x',y')$$ to $$(x',0)$$ and $$(x'',y'')$$ to $$(x'',0)$$ mapped back to $$D_{n}^{+}$$ by $$(\Psi _{n}^{+})^{-1}$$ give the required arcs. This construction gives a Runge family, and the proof is reduced to the proof in the noncritical case: First we extend the maps $$F_{n,1},F_{n,2}$$ continuously to maps from $$B\times (K_n\cup \gamma _{n}\cup \gamma ^{p}_{n}\cup \gamma ^{q}_{n})$$ such that the image of $$B\times (\gamma _{n}\cup \gamma ^{p}_{n}\cup \gamma ^{q}_{n})$$ lies in $$\{z\in \mathbb {C}:\Re z>n\}$$, and that the image of $$B\times \{p_{n+1}^{k_{n+1}}\}$$ and $$B\times \{q_{n+1}^{k_{n+1}}\}$$ lies in $$\{z\in \mathbb {C}:\Re z>n+1\}$$. The continuous extension to $$B\times (\gamma _{n}\cup \gamma ^{p}_{n}\cup \gamma ^{q}_{n})$$ with the image in $$\{z\in \mathbb {C}:\Re z>n\}$$ is possible by the property $$(d)_n$$ and since the former set is contractible. Now the construction can proceed similarly to the construction in the regular case, and here we explain the main differences: In the noncritical case, the functions $$\phi _{n,j}$$ determined boundary arcs $$(\alpha _\text {odd})_B$$ and $$(\alpha _\text {even})_B$$ such that $$\Re F_{n,1}>n$$ on $$(\alpha _\text {odd})_B$$, and $$\Re F_{n,2}>n$$ on $$(\alpha _\text {even})_B$$. In the critical case, we start at the point $$p_n^{k_n}$$ on $$bK_n$$ and move in the positive direction along $$k_n$$-th component of $$bK_n$$; we first get some arcs with alternating parity until we reach the point $$\varphi _n^{k_n}(\phi _{n,2m_n^{k_n}-1}^{k_n}(b))$$. These arcs determine the domains in $$D_n^+$$ that belong to $$D_\text {odd}$$, $$D_\text {even}$$ as before. Observe that for all $$b\in B$$ and $$x\in \varphi _n^{k_n}([\phi _{n,2m_n^{k_n}-1}^{k_n}(b),\pi ])\cup \gamma _n\cup \varphi _n^{k_n-1}([0, \phi _{n,2}^{k_n-1}(b)])=:I^+(b)$$ we have $$\Re F_{n,1}(b,x)>n$$. Therefore, the set $$I^+$$ can be seen as a part of $$(\alpha _\text {odd})_B$$, and the corresponding domain as a part of $$D_\text {odd}$$. As we move further along the boundary of the $$(k_n-1)$$-th component of $$bK_n$$ in the positive direction, from $$\varphi _n^{k_n-1}(\phi _{n,2}^{k_n-1}(b))$$ to $$\varphi _n^{k_n-1}(\phi _{n,2l_n^{k_n-1}}^{k_n-1}(b))$$ we obtain alternating arcs and domains as before, first we get some from $$D_n^+$$ and then some in $$D_n^-$$. Similarly to the above, we have for all $$b\in B$$ and $$x\in \varphi _n^{k_n-1}([\phi _{n,2l_n^{k_n-1}}^{k_n-1}(b),2\pi ])\cup \gamma _n\cup \varphi _n^{k_n}([\pi , \phi _{n,2m_n^{k_n}+1}^{k_n}(b)])=:I^-(b)$$ that $$\Re F_{n,2}(b,x)>n$$, and the set $$I^-$$ can be viewed as a part of $$(\alpha _\text {even})_B$$, and the corresponding domain as a part of $$D_\text {even}$$. As we move further along the boundary of the $$k_n$$-th component of $$bK_n$$, we get some arcs with alternating parity until we reach the starting point $$p_n^{k_n}$$. Then we proceed with the proof as in the noncritical case. On the right part of Figure 3, we denoted the arcs in $$(\alpha _\text {odd})_B$$ darker than the arcs in $$(\alpha _\text {even})_B$$.

**Fig. 4:**
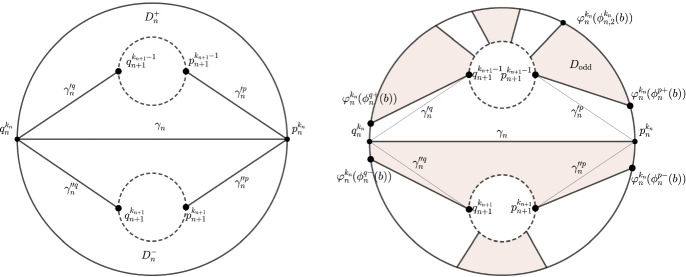
Critical case 2

In the second case, the endpoints of the arc $\gamma _n$ lie on the same component of $bK_n$. In this case, the number of components of $bK_{n+1}$ is one greater than the number of components of $bK_{n}$. By rearranging the notation, we may assume that the endpoints of $\gamma _n$ are $p^{k_n}_n$ and $q^{k_n}_n$. The set $K_{n+1}\setminus \text {Int}\,K_{n}$ is a union of a two connected domain $D_n$ in *X*, and perhaps a finite number of annuli, where the set $\gamma _n\cup (bK_n\cap D_n)$ separates the other boundary components of $D_n$. We can choose smooth arcs $\gamma '^{p}_{n}$, $\gamma '^{q}_{n}$, $\gamma ''^{p}_{n}$, $\gamma ''^{q}_{n}$ in $K_{n+1}\setminus \text {Int}\,K_{n}$ which intersect $bK_{n}$ and $bK_{n+1}$ transversally and only at their endpoints, such that the endpoints of $\gamma '^{p}_{n}$ are $p_{n}^{k_{n}}$ and $p_{n+1}^{k_{n+1}-1}$, the endpoints of $\gamma '^{q}_{n}$ are $q_{n}^{k_{n}}$ and $q_{n+1}^{k_{n+1}-1}$, the endpoints of $\gamma ''^{p}_{n}$ are $p_{n}^{k_{n}}$ and $p_{n+1}^{k_{n+1}}$, the endpoints of $\gamma ''^{q}_{n}$ are $q_{n}^{k_{n}}$ and $q_{n+1}^{k_{n+1}}$. Furthermore, we can achieve that arcs $\gamma _{n}$, $\gamma '^{p}_{n}$, $\gamma '^{q}_{n}$, $\gamma ''^{p}_{n}$, $\gamma ''^{q}_{n}$ intersect pairwise at most at their endpoints. We denote by $D_{n}^{+}$ the simply connected component of the set $D_{n}\setminus (\gamma '^{p}_{n}\cup \gamma '^{q}_{n})$. Assume that $D_{n}^{+}$ contains $\varphi _{n}^{k_{n}}((0,\pi ))$, the other case is symmetrical. Let $D_{n}^{-}$ be the simply connected component of the set $D_{n}\setminus (\gamma ''^{p}_{n}\cup \gamma ''^{q}_{n})$ which contains $\varphi _{n}^{k_{n}}((\pi ,2\pi ))$. See the left part of Figure 4.

There is a diffeomorphism $\psi _{n}^{+}$ from ${\bar{D}}_{n}^{+}$ (and $\psi _{n}^{-}$ from ${\bar{D}}_{n}^{-}$) to the unit square $[0,1]\times [0,1]$ in $\mathbb {R}^{2}$ that maps the arcs $\gamma '^{p}_{n}$, $\gamma '^{q}_{n}$ ($\gamma ''^{p}_{n}$ ,$\gamma ''^{q}_{n}$) to the vertical edges, and the arc $\varphi _{n}^{k_{n}}((0,\pi ))$, ($\varphi _{n}^{k_{n}}((\pi ,2\pi ))$) to the upper edge of the square. By the properties $(c)_{n}-(d)_{n}$ there are continuous functions $\phi ^{p\pm }_{n},\phi ^{q\pm }_{n}:B\rightarrow (0,2\pi )$ such that for each $b\in B$ we have $\phi ^{p+}_{n}(b)\in (0,\phi _{n,2}^{k_{n}}(b))$, $\phi ^{p-}_{n}(b)\in (\phi _{n,2l_n^{k_{n}}}^{k_{n}}(b),2\pi )$, $\phi ^{q+}_{n}(b)\in (\phi _{2m_n^{k_n}-1}^{k_n}(b),\pi )$, $\phi ^{q-}_n(b)\in (\pi ,\phi ^{k_n}_{2m_n^{k_n}+1}(b))$ and such that the restrictions of the maps $F_{n,1},F_{n,2}$ to the arcs $\varphi _{n}^{k_{n}}([0,\phi ^{p+}_{n}(b)])$, $\varphi _{n}^{k_{n}}([\phi ^{p-}_{n}(b),2\pi ])$, $\varphi _{n}^{k_{n}}([\phi ^{q+}_{n}(b),\pi ])$ and $\varphi _{n}^{k_{n}}([\pi ,\phi ^{q-}_{n}(b)])$ map into $\{z\in \mathbb {C}:\Re z>n\}$. Note that in this step we added 4 functions to the family $\phi ^{k_{n}}_{n,i}$. For every $b\in B$ and every $t\in [0,1]$ we get a segment from (*t*, 0) to $((1-t)\psi _n^+(\varphi _n^{k_n}(\phi ^{p+}_n(b)))+t\psi _n^+(\varphi _n^{k_n}(\phi ^{q+}_n(b))),1)$ in the unit square, and by pushing back with $(\psi _n^+)^{-1}$ we obtain a family of arcs in ${\bar{D}}_n^+$ that correspond to the union of radial line segments (and similarly for ${\bar{D}}_n^-$). In particular, for $t=0$ we get arc from $\varphi _n^{k_n}(\phi ^{p+}_n(b))$ to $p_{n+1}^{k_{n+1}-1}$, and for $t=1$ we get the arc from $\varphi _n^{k_n}(\phi ^{q+}_n(b))$ to $q_{n+1}^{k_{n+1}-1}$. Next, we explain how we divide the domain $D_{n}$ into domains $D_\text {odd}$ and $D_\text {even}$ which reduces the proof to the proof in the noncritical case (see the right part of Figure 4).

We start with the point $\varphi _n^{k_n}(\phi _n^{p+}(b))$ and move in the positive direction along $k_{n}$-th component of $bK_{n}$. We get arcs with alternating parity until we reach the point $\varphi _{n}^{k_{n}}(\phi _{n}^{q+}(b))$ determining the domains which alternately belong to $D_\text {odd}$, $D_\text {even}$. For $b\in B$ and $x\in \varphi _n^{k_{n}}([\phi _{n}^{q+}(b),\pi ])\cup \gamma _n\cup \varphi _n^{k_{n}}([0,\phi _{n}^{p+}(b)])=:I^{+}(b)$ it holds that $\Re F_{n,2}(b,x)>n$, thus, $I^+$ can be taken as a part of $\alpha _\text {even}$ and the corresponding domain as a part of $D_\text {even}$. For $b\in B$ and $x\in \varphi _n^{k_n}([\pi ,\phi _n^{q-}(b)])\cup \gamma _n\cup \varphi _n^{k_n}([\phi _n^{p-}(b),2\pi ])=:I^{-}(b)$ we have that $\Re F_{n,1}(b,x)>n$, thus, $I^{-}$ can be taken as a part of $\alpha _\text {odd}$ and the corresponding domain as a part of $D_\text {odd}$. From the point $\varphi _n^{k_n}(\phi _n^{q-}(b))$ we move in the positive direction along $bK_n$ until we reach the point $\varphi _n^{k_n}(\phi _n^{p-}(b))$ and again the points $\varphi _n^{k_n}(\phi _{n,i}^{k_n}(b))$ determine the arcs with alternating parity. Again, this reduces the construction to the noncritical case, which completes the proof. $\square$

### Proof of Theorem 1.3

In the proof of Theorem 1.1, we constructed a continuous map $F:B\times X\rightarrow \mathbb {C}^{2}$ such that for every $b\in B$ the map $F(b,\cdot ):(X,J_b)\rightarrow \mathbb {C}^{2}$ is proper holomorphic, and, furthermore, for every $b\in B$, $\max \{ \Re F_1(b,\cdot ),\Re F_2(b,\cdot )\}$ goes to infinity as we leave any compact set of *X*, which implies that the map $(\Re F_1,\Re F_2)(b,\cdot ):(X,J_b)\rightarrow \mathbb {R}^2$ is proper harmonic. $\square$
